# A biochemical framework for anaerobic oxidation of methane driven by Fe(III)-dependent respiration

**DOI:** 10.1038/s41467-018-04097-9

**Published:** 2018-04-24

**Authors:** Zhen Yan, Prachi Joshi, Christopher A. Gorski, James G. Ferry

**Affiliations:** 1 Department of Biochemistry and Molecular Biology, Penn State, University Park, PA 16801 USA; 2 Department of Civil and Environmental Engineering, Penn State, University Park, PA 16801 USA

## Abstract

Consumption of methane by aerobic and anaerobic microbes governs the atmospheric level of this powerful greenhouse gas. Whereas a biochemical understanding of aerobic methanotrophy is well developed, a mechanistic understanding of anaerobic methanotrophy has been prevented by the unavailability of pure cultures. Here we report a biochemical investigation of *Methanosarcina acetivorans*, a methane-producing species capable of anaerobic methanotrophic growth dependent on reduction of Fe(III). Our findings support a pathway anchored by Fe(III)-dependent mechanisms for energy conservation driving endergonic reactions that are key to methanotrophic growth. The pathway is remarkably similar to pathways hypothesized for uncultured anaerobic methanotrophic archaea. The results contribute to an improved understanding of the methane cycle that is paramount to understanding human interventions influencing Earth’s climate. Finally, the pathway enables advanced development and optimization of biotechnologies converting methane to value-added products through metabolic engineering of *M. acetivorans*.

## Abstract (web summary)

The unavailability of pure cultures has prevented a mechanistic understanding of anaerobic methanotrophy. Here the authors report a biochemical investigation of *Methanosarcina acetivorans* that supports a pathway anchored by Fe(III)-dependent mechanisms for energy conservation and driving endergonic reactions.

## Introduction

The production and consumption of methane is a component of the global carbon cycle. An end product of the anaerobic decomposition of biomass, nearly one billion tons of methane is produced each year in diverse anaerobic habitats of the Earth’s terrestrial biosphere^1^. Methane is also produced in anaerobic marine sediments and released from vast reservoirs of gas hydrates (5.0 × 10^5^–1.0 × 10^7^ Tg)^2^. Methane is an important greenhouse gas nearly 20-fold more potent than CO_2_^3^. Fortunately, release to the atmosphere is mitigated by the aerobic and anaerobic oxidation of methane or assimilation into cell biomass by microbes that ultimately control the Earth’s climate^4^.

The anaerobic oxidation of methane (AOM) requires reduction of electron acceptors (Fe(III), Mn(IV), nitrate or sulfate) to be thermodynamically favorable^5,6^. Until recently, it was thought that AOM in marine sediments required a symbiosis of anaerobic methanotrophic archaea (ANME) and sulfate-reducing species for which the latter utilizes reductant produced by the former to make the overall reaction thermodynamically favorable. However, the artificial electron acceptor AQDS (9,10-anthraquinone-2,6-disulfonate), a surrogate for humic substances in the environment, decouples methane oxidation from sulfate reduction in marine sediments presenting the possibility of independent respiratory AOM and growth of usually syntrophic ANME-2^7,8^. Indeed, respiratory AOM is in accordance with the occurrence of solitary ANME in microbial mats and sediments from diverse anaerobic environments^9^. Furthermore, incubations of environmental samples with Fe(III)-citrate yielded biosynthetic activity exclusive to uncultured ANME-2c and other ANME-2 cells^7^. These results inspired the hypotheses that symbiotic associations of ANME and sulfate-reducing species evolved from methanogenic species that first acquired the ability to conserve energy by oxidizing methane and reducing metal oxides^7^. Fe(III)-dependent AOM reported for a culture enriched in *‘Candidatus* Methanoperedens nitroreducens’ presents the possibility of Fe(III)-dependent respiratory growth although not yet documented^10^. AOM dependent on reduction of Fe(III) is of particular biogeochemical interest. Indeed, it is proposed that if only a small percentage of current global Mn(IV) and Fe(III) influx is used for AOM, it has the potential to consume a large amount of methane^11^. It is also proposed that Fe(III)-dependent AOM was largely responsible for oxidizing the entirety of methane produced on early Earth prior to the advent of oxygen^11^.

The prospect that ANME are capable of independent AOM and growth by Fe(III) respiration profoundly changes current views of AOM and iron cycling in Nature, prompting further investigation. It is conjectured that ANME grow by reverse methanogenesis based on environmental metagenomic and metatranscriptomic analyses of sediments^5,6^. However, reversal and growth requires mechanisms for energy conservation and overcoming endergonic reactions yet to be investigated biochemically^5,6^. Clearly, biochemical approaches with pure cultures are necessary to obtain a rigorous understanding of AOM. Although discovered nearly four decades ago, the unavailability of pure cultures has prevented biochemical investigations of AOM. However, a culture enriched in *‘Candidatus* Methanoperedens nitroreducens’ is capable of AOM dependent on reduction of nitrate-, nitrite- or Fe(III)^10,12^. Furthermore, *Methanosarcina acetivorans* strain C2A is capable of trace methane oxidation (TMO) defined as reverse methanogenesis during net methane production from growth substrates in the absence of external electron acceptors^6,13,14^. TMO contrasts with AOM which is independent of methanogenesis and requires electron acceptors with or without a syntrophic partner. More recently, Fe(III)-dependent AOM was documented for a strain of *M. acetivorans* engineered to produce methyl-coenzyme M methyl reductase (Mcr) derived from ANME-1 sediments^15^. Thus, *M. acetivorans* has emerged as a model for advancing a biochemical understanding of AOM. *M. acetivorans* is also recognized as instrumental in development of biomanufacturing processes converting methane into value-added products^16–18^. Here we report a biochemical investigation of wild-type *M. acetivorans* that supports an AOM pathway anchored by Fe(III)-dependent respiration generating ion gradients that supply the energy to drive endergonic reactions essential for AOM and growth. The results provide a deeper mechanistic understanding of AOM and iron cycling in Nature, and a guide for optimization of methane-based biotechnologies.

## Results

### Biochemistry of *M. acetivorans* resembles proposals for AOM

Mechanisms for energy conservation and driving endergonic reactions are key to reversing methanogenesis and methanotrophic growth (AOM). Based on environmental metagenomic and transcriptomic analyses, AOM pathways are proposed for ANME that include membrane-bound components involved in energy conservation (Rnf and Fpo complexes), electron transport (CoMS-SCoB heterodisulfide reductase (HdrDE); multi-heme *c*-type cytochromes (MHC); and methanophenazine (MP)) and methyl transfer (methyl-tetrahydrosarcinapterin:coenzyme M methyltransferase (Mtr))^5,6,19–21^. These components are essential to biochemically characterized methanogenic pathways in *M. acetivorans* strain C2A. Thus, the components were investigated in strain C2A to provide a biochemical understanding of roles in AOM of ANME.

### Driving endergonic methane oxidation to the methyl level

**Fig. 1:**
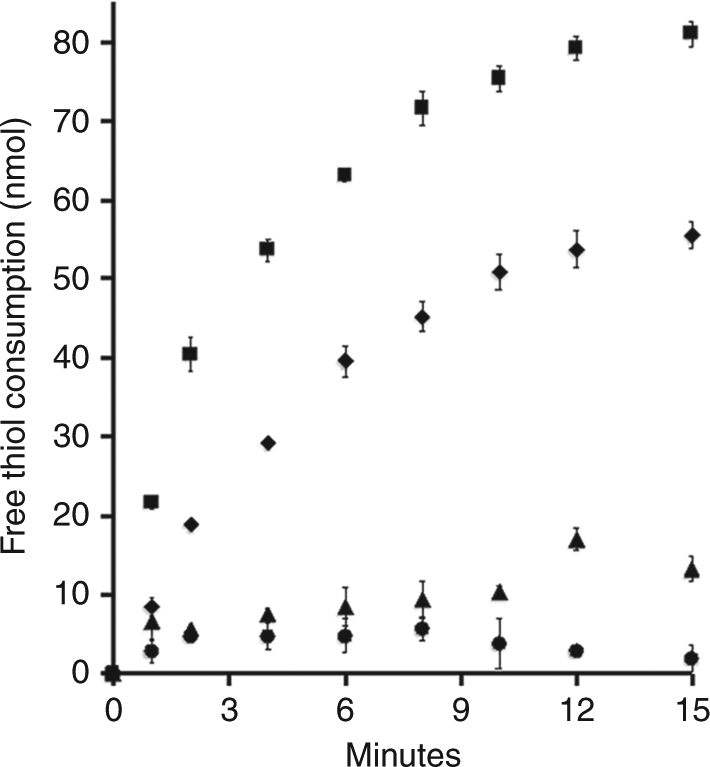
Reverse heterodisulfide reductase activity of everted membrane vesicles dependent on Fe(III) as electron acceptor. The standard reaction mixture (1.0 ml) contained 1.0 mM each of HSCoM (coenzyme M) and HSCoB (coenzyme B) in 50 mM Tris (pH 8.0) and 1.0 atmosphere Ar. The reaction was started by adding everted membrane vesicles (0.5 mg protein) prepared from acetate-grown cells that were empty (filled circles), loaded with Fe(III) (filled triangles), loaded with AQDS (9,10-anthraquinone−2,6-disulfonate) (filled diamonds) or loaded with Fe(III) plus AQDS (filled squares). Vesicles loaded with Fe(III) plus AQDS produced 88.4 ± 5.4 nmol Fe(II) at 15 min consistent with the expected stoichiometry. No free thiol consumption was detected (limit = 0.5 nmol) if only HSCoM or only HSCoB replaced HSCoM plus HSCoB. Data points are the mean of three biological replicates for which the standard deviation is shown by error bars

The finding that both acetate and CO_2_ are produced during TMO and AOM by *M. acetivorans* indicates a dependence on reversal of acetoclastic and CO_2_-reducing methanogenic pathways, albeit with essential modifications^13–15^. The first two reactions required for reversal are methane oxidation yielding methyl-coenzyme M (CH_3_-SCoM) and transfer of the methyl group to tetrahydrosarcinapterin (H_4_SPT). Both reactions are endergonic (Supplementary Table 1). The coenzyme M (HSCoM) and coenzyme B (HSCoB) products are oxidized to the heterodisulfide (CoMS-SCoB) which is the electron acceptor for the methane oxidation reaction yielding CH_3_-SCoM and HSCoB. The exergonic oxidation of HSCoM and HSCoB coupled to Fe(III) reduction is a potential mechanism driving the endergonic methane oxidation and methyl transfer reactions (Supplementary Table 1). This was tested by monitoring HSCoM and HSCoB oxidation by everted membrane vesicles prepared from acetate-grown *M. acetivorans*. The initial rate of free thiol consumption by vesicles loaded with S-layer-permeable Fe(III) citrate and AQDS (46.2 nmol/min/mg vesicle protein) is 2.5- and 12.1-fold greater than the rate by vesicles loaded with only AQDS (18.5 nmol/min/mg vesicle protein) or only Fe(III) citrate (3.8 nmol/min/mg vesicle protein) (Fig. 1). As HdrDE is the only membrane-bound heterodisulfide reductase^22–24^, the results show that HdrDE oxidizes HSCoM/HSCoB with transfer of electrons to Fe(III) mediated by AQDS with the potential for product removal driving the endergonic oxidation of methane and transfer of the methyl group to H_4_SPT.

### Generation of a sodium gradient

Two membrane-bound complexes generate a Na^+^ gradient (high outside) in the acetoclastic pathway of *M. acetivorans*^22^. The Rnf complex catalyzes the vectorial translocation of Na^+^ generating a gradient (high outside) when oxidizing ferredoxin (Fdx_R_) with transfer of electrons to an electron transport chain culminating in reduction of CoMS-SCoB^25–27^. A Na^+^ gradient is also generated by the exergonic transfer of the methyl group from CH_3_-H_4_SPT to HSCoM by the methyltransferase (Mtr)^22^. A Rnf homolog plays a role in generating a Na^+^ gradient in the AOM pathway proposed for an uncultured ANME-2a organism based on the metagenome and transcriptome^6,19,21^. This known biochemistry raised the question of whether oxidation of Fdx_R_ by everted vesicles of *M. acetivorans* is coupled to reduction of Fe(III) that drives formation of a Na^+^ gradient of sufficient magnitude to reverse the exergonic methyl transfer catalyzed by Mtr necessary for reversal of the acetoclastic and CO_2_-reduction methanogenic pathways.

Fe(III)-loaded everted vesicles catalyze Fdx_R_:Fe(III) oxidoreductase (ORase) activity independent of AQDS and with rates similar to that of vesicles loaded with both AQDS and Fe(III), although sixfold greater than that of vesicles loaded with only AQDS (Supplementary Fig. 1). The rate of Fe(III)-dependent Fdx_R_ oxidation is fourfold less with vesicles prepared from methanol-grown cells, a result consistent with a role for the Rnf complex that is downregulated in methanol- vs. acetate-grown *M. acetivorans*^25,26,28^. Furthermore, Rnf is the only membrane-bound system capable of oxidizing Fdx_R_^23,25,27^. The results establish that oxidation of Fdx_R_ is coupled to the reduction of Fe(III) (Δ*E*^*o*^´ = 1.17 V) capable of generating a Na^+^ gradient driving reversal of methyl transfer by Mtr.

A multi-heme *c*-type cytochrome (MHC), abundant in membranes of acetate-grown *M. acetivorans*, is reduced with Fdx_R_ and re-oxidized by addition of Fe(III)-citrate independent of AQDS (Supplementary Fig. 2), indicating a role for MHC in Fdx_R_:Fe(III) ORase activity^26^. The failure of AQDS to enhance Fdx_R_:Fe(III) ORase activity in everted vesicles (Supplementary Fig. 1) indicates direct transfer of electrons from a membrane-bound electron carrier to soluble Fe(III) citrate. The MHC is a strong candidate as it is the direct electron acceptor of the Rnf complex that oxidizes Fdx_R_^25,27^. Whole cells reduce insoluble ferrihydrite with CO as the electron donor for which the initial rate is stimulated fourfold in the presence of AQDS, albeit fourfold less than with Fe(III) citrate (Supplementary Fig. 3). This result indicates that AQDS is essential to mediate electron transfer to insoluble Fe(III) oxides.

**Fig. 2:**
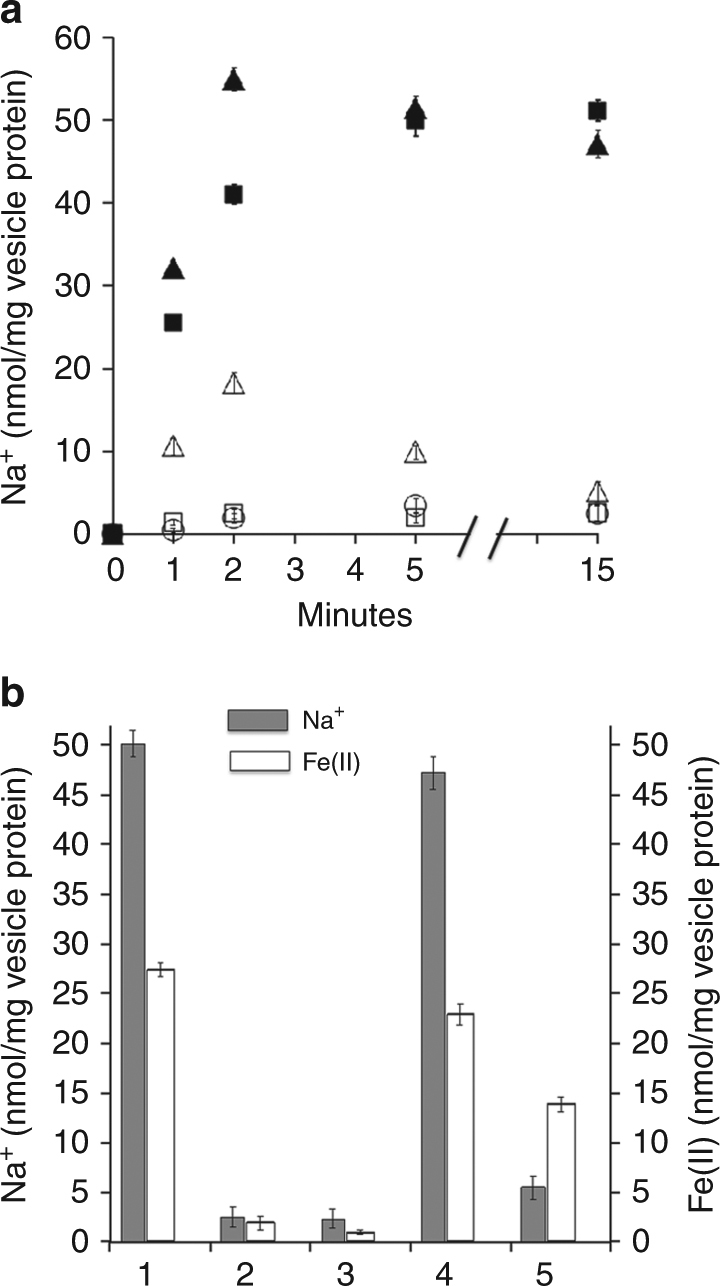
Accumulation of ^22^Na^+^ in the lumen of everted membrane vesicles dependent on ferredoxin:Fe(III) oxidoreductase activity. The standard reaction mixture (1.0 ml) contained Fe(III)-loaded vesicles (1.0 mg protein) from acetate-grown cells, 5.0 μM Fdx (ferredoxin), 0.5 mM NaCl, 0.5 μCi/ml ^22^NaCl (carrier free), 25 mM MgSO_4_, 0.4 M sucrose and 10 mM dithiothreitol in 50 mM Tris (pH 8.0) and 1.0 atmosphere of CO. Reactions were initiated by addition of CO dehydrogenase/acetyl-CoA synthase (0.1 mg). **a** Time course of accumulation. Data points are the mean of three biological replicates for which the standard deviation is shown by error bars. Standard reaction mixture (filled squares), minus Fdx (empty squares), with empty vesicles (empty circles), plus 10 µM carbonyl cyanide *m*-chlorophenyl hydrazine (CCCP) (filled triangles), plus 10 μM ETH157 (empty triangles). No accumulation occurred if N_2_ replaced CO. **b** Sodium and Fe(II) accumulation in the lumen of vesicles at the 15-min time point shown in **a**. The standard reaction mixtures were modified as indicated for each bar: (1) standard, no modification, (2) minus Fdx, (3) empty vesicles, (4) plus 10 μM CCCP, (5) plus 10 μM ETH157. Bars are the mean of three biological replicates for which the standard deviation is shown by error bars

The Fdx_R_:Fe(III) ORase activity of Fe(III) citrate-loaded everted vesicles is stimulated fourfold by the addition of NaCl to the reaction mixture that is concentration dependent with a *K*_m_ of ~1.0 mM (Supplementary Fig. 1). The stimulation is confirmed with vesicles loaded with both AQDS and Fe(III) citrate. The results are consistent with Na^+^ translocation into the lumen driven by electron transport. The apparent translocation of Na^+^ was further investigated with ^22^Na^+^ that rapidly accumulates in everted vesicles (25 nmol/mg protein/min) dependent on Fe(III) and Fdx_R_ (Fig. 2a). The Na^+^ ionophore ETH157 abolishes accumulation. These results establish that membranes translocate Na^+^ coupled to electron transport from Fdx_R_ to Fe(III). The protonophore CCCP (carbonyl cyanide *m*-chlorophenyl hydrazine) has no effect on the rate of Na^+^ accumulation which precludes that Na^+^ translocation is a secondary effect driven by a primary H^+^ gradient combined with a Na^+^/H^+^ antiporter. Accumulation of Fe(II) in vesicles correlates with accumulation of ^22^Na^+^ for each experimental condition except as expected for addition of ETH157 (Fig. 2b). Vesicles loaded with Fe(III) completely oxidize limiting amounts of fully reduced Fdx_R_^2-^ (2.5 nmol) and accumulate a total of 4.7 ± 0.4 (*n* = 3) nmol Fe(II) consistent with the expected stoichiometry. The ratio of ^22^Na^+^/Fe(II) is 1.8 ± 0.2 indicating translocation of two Na^+^/electron transported. Generation of a Na^+^ gradient by Rnf and the highly exergonic Fe(III)-dependent oxidation of the reaction product HSCoM by HdrDE are potential mechanisms for driving the endergonic methyl transfer reaction by Mtr.

### Driving endergonic methyl transfer

**Fig. 3:**
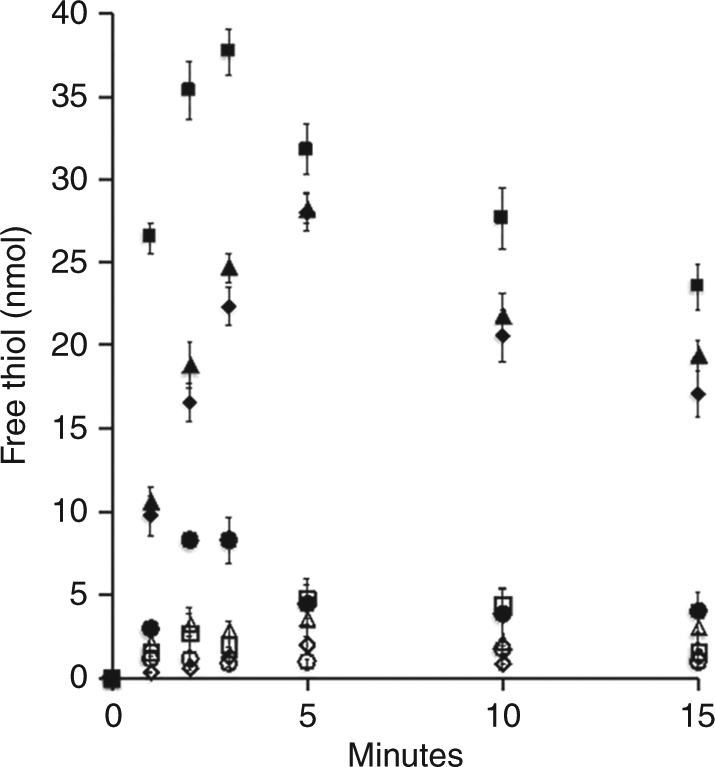
Reverse methyltransferase activity in the direction of CH_3_-tetrahydrofolate production driven by a sodium gradient dependent on reduced ferredoxin:Fe(III) oxidoreductase activity. The standard reaction mixture (1.0 ml) contained Fe(III)-loaded vesicles (1.0 mg protein), 2.0 mM CH_3_-SCoM (methyl-coenzyme M), 2.0 mM THF (tetrahydrofolate), 5.0 µM Fdx (ferredoxin), 25 mM MgSO_4_, 0.4 M sucrose and 0.5 mM residual NaCl in 20 mM Tris (pH 7.0) and 1.0 atmosphere of CO. Reactions were initiated with CO dehydrogenase/acetyl-CoA synthase (0.1 mg). Standard reaction mixture (filled diamonds), plus 1 mM ATP (filled squares), plus 10 µM carbonyl cyanide *m*-chlorophenyl hydrazine (CCCP) (filled triangles), plus 10 µM ETH157 (filled circles), minus Fdx (empty squares), with empty vesicles (empty diamonds), minus CH_3_-SCoM (empty circles), minus THF (empty triangles). Data points are the mean of three biological replicates for which the standard deviation is shown by the error bars

It was next asked if the Na^+^ gradient generated by Fdx_R_:Fe(III) ORase activity is of sufficient magnitude to drive the endergonic methyl transfer from CH_3_-SCoM to H_4_SPT by Mtr. Tetrahydrofolate (THF), a functional homolog of H_4_SPT, was used in the experiments^29^. Membranes catalyze methyl transfer in the thermodynamically favorable direction of CH_3_-SCoM synthesis with the methyl group donated by CH_3_-THF (Supplementary Fig. 4). The maximum initial rate of activity (180.8 ± 4.2 nmol/min/mg protein) is dependent on carbon monoxide (CO) and adenosine triphosphate (ATP), a result characteristic of reductive activation for Mtr of *Methanosarcina* species^29^. Everted membrane vesicles catalyze Fdx- and Fe(III)-dependent methyl transfer from CH_3_-SCoM to THF with an initial rate of 9.8 ± 1.2 nmol/min/mg protein that is abolished by the Na^+^ ionophore ETH157 (Fig. 3). The presence of ATP stimulates the initial rate to 26.5 ± 0.9 nmol/min/mg protein consistent with results reported for Mtr^29^. The results demonstrate that the Na^+^ gradient generated by Fdx_R_:Fe(III) ORase activity is of sufficient magnitude to drive the endergonic methyl transfer from CH_3_-SCoM to H_4_SPT.

### Conservation of energy

The mechanisms by which energy is conserved in ATP for growth of ANME is of fundamental importance in addition to mechanisms driving essential endergonic reactions. Substrate-level phosphorylation catalyzed by acetate kinase is a potential mechanism for ATP synthesis when considering acetate is a product of AOM by *M. acetivorans*^15^. Another possibility is ATP synthesis catalyzed by the ATP synthase which is dependent on both Na^+^ and H^+^ gradients^30^. This mechanism requires generation of a H^+^ gradient to accompany the Na^+^ gradient generated by Fdx_R_:Fe (III) ORase activity. The membrane-bound Fpo complex catalyzes a vectorial translocation of H^+^ generating a gradient (high outside) by oxidizing coenzyme F_420_H_2_ with transfer of electrons to HdrDE (F_420_H_2_:CoB-S-SCoM ORase activity)^31^. Based on metagenomic and transcriptomic analyses, Fpo homologs are proposed to play a role in generating H^+^ gradients in AOM pathways based on metagenomic and metatranscriptomic analyses of ANME^5,19–21,32^ which prompted asking if the oxidation of F_420_H_2_ by the Fpo of *M. acetivorans* is coupled to reduction of Fe(III) (F_420_H_2_:Fe(III) ORase activity) that also generates a H^+^ gradient. Fpo activity is substantial in methanol-grown *M. acetivorans*^28,33^ from which Fe(III) citrate-loaded everted membrane vesicles were prepared to evaluate F_420_H_2_:Fe(III) ORase activity. The vesicles catalyze Fe(III)-dependent F_420_H_2_ oxidation (Supplementary Fig. 5) with a sevenfold stimulation of the initial rate upon addition of the protonophore CCCP, indicating electron transport coupled to H^+^ translocation. The rate is fourfold less with vesicles from acetate-grown cells corresponding to fivefold less Fpo, a result supporting a role for Fpo in Fe(III)-dependent F_420_H_2_ oxidation. Vesicles accumulate a total of 11.2 ± 1.1 nmol (*n* = 3) Fe(II) in reaction mixtures containing 5.0 nmol F_420_H_2_ that is oxidized to completion (not shown), a result expected for the obligate two-electron carrier^34^. Negligible Fe(II) is produced in the absence of F_420_H_2_ or Fe(III).

**Fig. 4:**
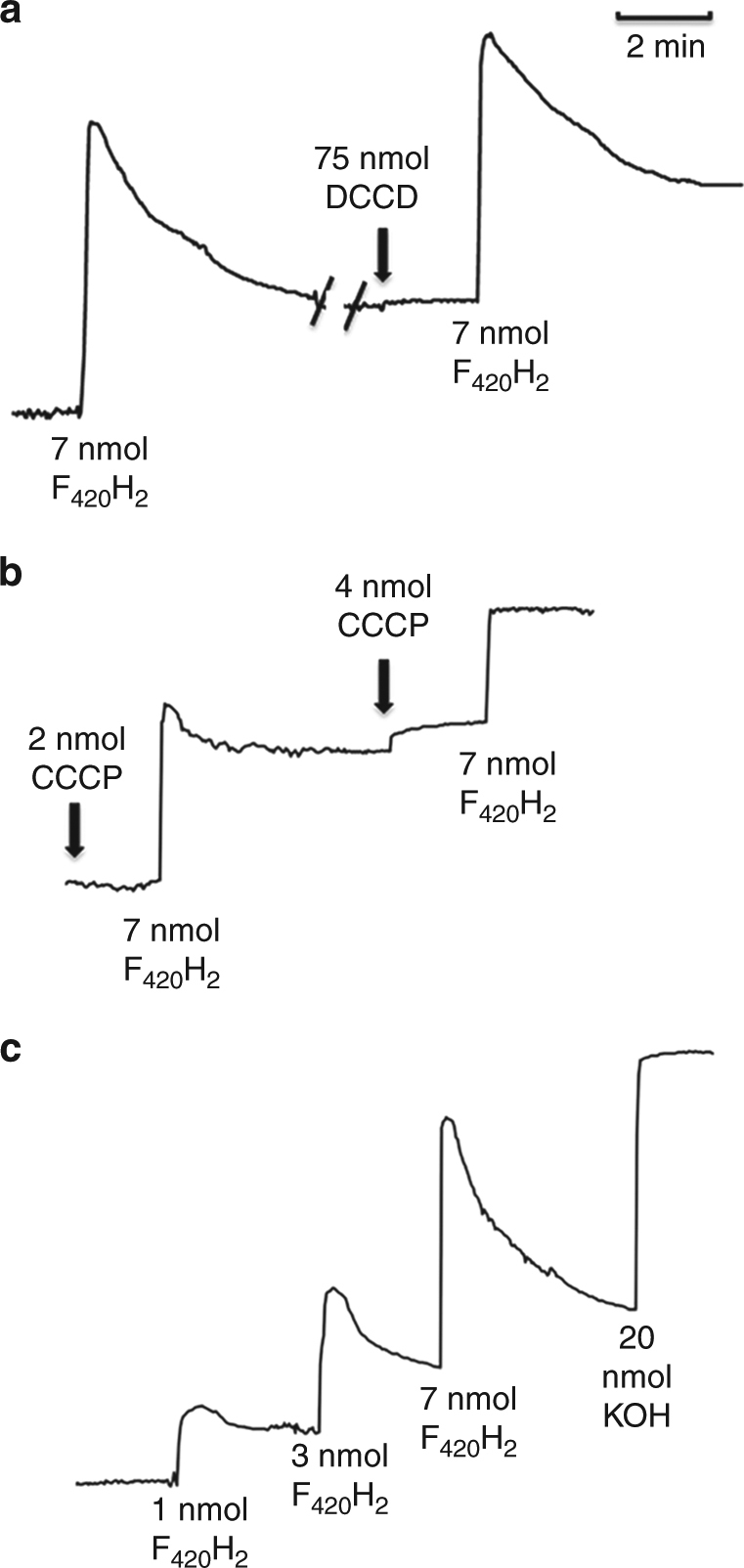
Proton uptake in everted membrane vesicles dependent on coenzyme F_420_H_2_:Fe(III) oxidoreductase activity. The reaction mixtures (2.0 ml) contained 40 mM KSCN, 0.4 M sucrose, 10 mM dithiothreitol and Fe(III)-loaded vesicles (0.5 mg protein) from methanol-grown cells in 2 mM potassium phosphate buffer (pH 7.0) and the indicated final concentration of reduced coenzyme F_420_ (F_420_H_2_). Additional reagents were added as indicated with final concentrations shown. **a** Effect of dicyclohexylcarbodiimide (DCCD). **b** Effect of carbonyl cyanide *m*-chlorophenyl hydrazine (CCCP). **c** Effect of increasing concentrations of F_420_H_2_. The difference between the starting baseline and the final baseline represents alkalinization of the medium dependent on additions

The finding that CCCP stimulates F_420_H_2_-dependent reduction of Fe(III) prompted experiments to determine if the highly exergonic electron transport (Δ*E*^*o*^´ = 1.15 V) is coupled to vectorial pumping of H^+^ from the medium into the lumen of everted vesicles loaded with Fe(III) citrate (Fig. 4). Addition of F_420_H_2_ produces rapid alkalinization of the medium followed by gradual acidification towards stabilization of the pH (Fig. 4a). The ATPase inhibitor, *N*,*N*’-dicyclohexylcarbodiimide (DCCD), has no effect on H^+^ translocation, ruling out a role for ATP-driven translocation. Negligible alkalinization and re-acidification is observed with vesicles devoid of Fe(III) or when F_420_ replaces F_420_H_2_ (not shown). Increased concentrations of the protonophore CCCP (carbonyl cyanide *m*-chlorophenyl hydrazine) correlate with decreased F_420_H_2_-dependent alkalinization (Fig. 4b). The results demonstrate that the electron transport is coupled to translocation of H^+^ from the medium into the lumen. Increased alkalinization correlates with increased amounts of F_420_H_2_ from which it is calculated that 2.1 H^+^ enters into the lumen per F_420_H_2_ oxidized (Fig. 4c). However, the results indicate translocation of 4 H^+^ when considering 2 H^+^ are produced outside the vesicle upon oxidation of F_420_H_2_. The result is consistent with the stoichiometry predicted for the F_420_H_2_:CoMS-SCoB ORase activity of *Methanosarcina* species^35^. Although all the electron transfer components have yet to be determined, the results establish that F_420_H_2_:Fe(III) ORase activity is coupled to generation of a H^+^ gradient with the potential to assist in ATP synthesis by the Na^+^- and H^+^-dependent ATP synthase.

### Proposed pathway of Fe(III)-dependent AOM and methanotrophic growth

**Fig. 5:**
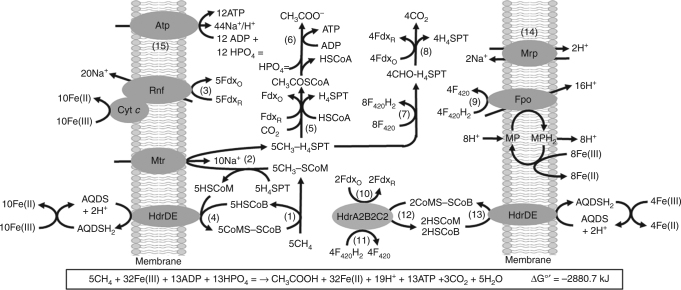
Pathway proposed for Fe(III)-dependent methane oxidation and conservation of energy by *M. acetivorans*. Enzymes not discussed in the text: CO dehydrogenase/acetyl-CoA synthase (Rxn. 5); acetate kinase and phosphotransacetylase (Rxn. 6); coenzyme F_420_ (F_420_)-dependent methylene-tetrahydrosarcinapterin (H_4_SPT) reductase, F_420_-dependent methylene-H_4_SPT dehydrogenase, methenyl-H_4_SPT cyclohydrolase, and formylmethanofuran:H_4_SPT formyltransferase (Rxn. 7); formylmethanofuran dehydrogenase (Rxn. 8). MP, methanophenazine

Acetate and CO_2_ are products of Fe(III)-dependent AOM by *M. acetivorans* indicating reversal of both acetoclastic and CO_2_-reducing methanogenic pathways^15^. Figure 5 shows a pathway proposed for soluble Fe(III)-dependent AOM by *M. acetivorans* based on results presented here and characterized components of the well-established acetoclastic and CO_2_-reducing methanogenic pathways of *M. acetivorans*^23,25–28,33,36,37^. As the outer S-layer of *M. acetivorans* contains pores of sufficient size to permit passage of Fe(III)-citrate, results obtained with everted vesicles can be extrapolated to whole cells^38^.

In the proposed pathway (Fig. 5), methane is assumed oxidized by the methyl-coenzyme M methyl reductase (Mcr) (Rxn. 1) based on TMO and AOM by *M. acetivorans* that requires Mcr activity^13–15^. The methyl group of CH_3_-SCoM is then transferred to H_4_SPT by the membrane-bound methyltransferase (Mtr) (Rxn. 2). The results presented show the endergonic reaction is driven by the Na^+^ gradient generated with Fdx_R_:Fe(III) ORase activity catalyzed by the Rnf complex (Rxn. 3)^23,27^. The results also indicate that MHC is the direct electron donor to Fe(III), analogous to reduction of soluble and insoluble forms of Fe(III) by the MHC of Fe(III) reducing microbes such as *Shewanella oneidensis*^39,40^.

The HSCoM and HSCoB products of reactions 1 and 2 are oxidized to regenerate CoMS-SCoB essential for Mcr-catalyzed methane oxidation (Rxn. 1). The results presented indicate the oxidation is catalyzed by the membrane-bound HdrDE (Rxn. 4) dependent on AQDS-mediated reduction of Fe(III). The endergonic oxidation of methane (Rxn. 1) is driven by exergonic reaction 4 coupled with reaction 2 driven by exergonic reaction 3 (Supplementary Table 1). The path of AQDS-dependent electron transport from HdrDE to Fe(III) is unknown. MP is a quinone-like, membrane-intrinsic, electron carrier that donates to cytochrome *b* in all pathways of methanogenesis by *M. acetivorans*^23,27^. Although reduction of MP by cytochrome *b* is expected, MPH_2_ is also expected to directly reduce Fe(III) analogous to abiotic reduction of AQDS^41,42^ obviating a requirement for AQDS in contrast to that observed. Thus, a more plausible pathway involves a role for AQDS accepting electrons from cytochrome *b* of the membrane-intrinsic HdrE and AQDS abiotically reducing Fe(III) (Fig. 5).

It is proposed that the methyl group of CH_3_-H_4_SPT enters branched pathways for production of acetate (Rxns. 5–6) and oxidation to CO_2_ (Rxns. 7–8). Transfer of electrons to Fe(III) in the CO_2_ oxidation branch is essential for the overall pathway to be thermodynamically favorable (Supplementary Table 1). The acetate branch is included based on previous findings that acetate is a product of Fe(III)-dependent AOM by *M. acetivorans*^15^. The exergonic reactions leading to acetate are experimentally validated for the pathway of CO-dependent growth by *M. acetivorans*^33^. Reactions proposed for oxidation of the methyl group of CH_3_-H_4_SPT to CO_2_ (Rxns. 7 and 8) are components of the CO_2_-reduction pathway of *M. acetivorans*^33^. The pathway (Fig. 5) proposes that the F_420_H_2_ produced is re-oxidized with transfer of electrons to Fe(III) by two possible mechanisms. The first is by exergonic F_420_H_2_:Fe(III) ORase activity coupled to the generation of a H^+^ gradient (Rxn. 9) involving Fpo that is also essential to the CO_2_-reduction pathway of *M. acetivorans*^33^. Although the path of electron transfer from F_420_H_2_ to Fe(III) is unknown, failure of AQDS to stimulate F_420_H_2_:Fe(III) ORase activity indicates electron transfer from Fpo to Fe(III) is independent of AQDS and HdrDE in contrast to electron transport from HdrDE to Fe(III). A role for MP is possible as it is the electron acceptor for Fpo^23,35^. It is proposed that the MPH_2_ generated is oxidized by Fe(III) contributing to the H^+^ gradient (Fig. 5, Rxn. 9). Alternatively, F_420_H_2_ may be oxidized by the recently characterized electron bifurcating HdrA2B2C2 of *M. acetivorans* (Rxn. 10) with transfer of electrons to Fdx_O_ (Rxn. 11) and CoMS-SCoB (Rxn. 12)^43^. Oxidation of HSCoM and HSCoB (Rxn. 13) is postulated to be accomplished by HdrDE with exergonic transfer of electrons to Fe(III) dependent on AQDS analogous to reaction 4.

Finally, it is proposed that the ratio of acetate/CO_2_ produced is flexible and governs the ratio of Na^+^/H^+^ gradients requiring the multi-subunit Na^+^/H^+^ antiporter Mrp (Rxn. 14) to adjust the Na^+^/H^+^ ratio optimal for the ATP synthase shown previously to be dependent on both Na^+^ and H^+^ (Rxn. 15)^30,44^.

## Discussion

The proposed biochemical-based pathway elevates AOM by *M. acetivorans* to a fundamental mechanistic level of understanding. The pathway is essentially a reversal of the biochemically characterized acetoclastic and CO_2_-reduction methanogenic pathways, albeit dependent on reduction of Fe(III). The results reveal the role Fe(III) plays in energy conservation and driving endergonic reactions that support methanotrophic growth^15^. Abundant methane is targeted for development of recently advocated biomanufacturing processes converting it to liquid biofuels and value-added products^16^. Thus, the pathway provides a guide for optimization of existing, and development of advanced, biotechnologies facilitated by the timely announcement of a cas9-mediated genome editing system for facile metabolic engineering of *M. acetivorans*^16–18,45–47^.

The proposed *M. acetivorans* pathway is remarkably similar to the pathway proposed for ANME-2a based on the single-aggregate genome derived from integrated environmental metagenomic and transcriptomic analyses of a marine environment^21^. The *M. acetivorans* and ANME-2a pathways both include Rnf, Fpo, MHC, HdrDE and CO dehydrogenase/acetyl-CoA synthase for which the homologs show robust amino acid sequence identities (Supplementary Table 2). The results presented, showing generation of Na^+^ and H^+^ ion gradients by Rnf and Fpo of *M. acetivorans*, validate the same roles for Rnf and Fpo in pathways proposed for uncultured ANME^20,21,32^. The pathways for *M. acetivorans*, ANME-1, ANME-2a and ANME-2d include acetate as a product^12,21,32^. Furthermore, analysis of methane seep sediments show robust ^13^C depletion in acetate with δ^13^C values near −90%^48^. These results suggest that the proposed AOM pathway of *M. acetivorans* is largely representative of ANME, further strengthened by data showing ANME-1, ANME-2d and ANME-2a are phylogenetically related to *Methanosarcina* species^10,20,21,49^. The one exception is Mcr of wild-type *M. acetivorans* which could explain why only the engineered strain containing Mcr from ANME-1 is capable of Fe(III)-dependent AOM^15^. No other genetic alterations were imposed on the engineered strain ensuring that mechanisms for Fe(III)-dependent energy conservation and driving endothermic reactions are identical to those investigated in the wild type. Although TMO by wild-type *M. acetivorans* indicates that Mcr catalyzes the reverse reaction analogous to that of *Methanothermobacter marburgensis*^50^, bias in the direction of methane formation may be a kinetic block to initiating AOM as opposed to a more favorable rate of methane oxidation for Mcr of ANME.

The similarity in pathways for *M. acetivorans* and proposed for ANME-2a strengthen the prospect of Fe(III)-dependent AOM and respiratory growth by uncultured ANME. Importantly, the roles shown for Rnf, MHC, Fpo and HdrDE in electron transfer to Fe(III) by *M. acetivorans* are also hypothesized for ANME-2 in transfer of electrons to metal oxides or the syntrophic partner^19^. The MHCs of ANME-2 are proposed to extend beyond the S-layer to facilitate the electron transfer. The results presented here indicate that soluble Fe(III) citrate is reduced by the MHC of *M. acetivorans*. It is conceivable that the MHC also plays a role in reducing insoluble forms of Fe(III). If so, either multiple MHCs extend to the outer S-layer of *M. acetivorans* or diffusible electron carriers such as low-molecular-mass humic acids or secreted flavins mediate electron transfer between the MHC and the S-layer^7,51^. The latter is consistent with the finding that AQDS, a surrogate for humics, stimulated ferrihydrite reduction in whole cells of *M. acetivorans*. Extension of MHCs to the outer S-layer is consistent with MHCs from ANME-2a and ANME-2d reported to be fused with a putative S-layer domain homologous to the S-layer protein of *M. acetivorans*^19^.

The pathway proposed for *M. acetivorans* is also consistent with the hypothesis that symbiotic associations of ANME and sulfate-reducing species evolved from methanogenic species that first acquired the ability to conserve energy by oxidizing methane coupled to reduction of metal oxides^7^. However, it is also possible that the ancestor common to *M. acetivorans* and ANME first performed AOM with a sulfate reducer and then switched to reduction of metal oxides. Unlike methanogenesis pathways, the proposed AOM pathway of *M. acetivorans* features multiple roles for heterodisulfide reductases (Fig. 5). Two types of heterodisulfide reductases are essential to all methanogenic pathways, the cytoplasmic HdrABC and the membrane-associated HdrDE^23,24^. The results presented here support a role for HdrDE coupled to AQDS-mediated reduction of Fe(III) that drives the endergonic methane oxidation and methyl transfer reactions essential to reversal. A role is proposed for the electron bifurcating HdrA2B2C2 in energy conservation when generation of Na^+^ and H^+^ gradients by Rnf and Fpo (Fig. 5, Rxns. 3 and 9) are limited by the availability of Fe(III). The HdrA2B2C2 plays this role by diverting electron flow from F_420_H_2_ to Fdx_O_ promoting acetate production and ATP synthesis by substrate-level phosphorylation (Fig. 5, Rxns. 5 and 6). Notably, the genome of the ANME ‘*Candidatus* Methanoperedens nitroreducens’ encodes a homolog of HdrA2 that is the flavin-containing electron-bifurcating subunit of HdrA2B2C2^43,52^.

A role for H_2_ in electron transport and energy conservation is unlikely for Fe(III)-dependent AOM. *M. acetivorans* is incapable of metabolizing H_2_ which contrasts with other species of *Methanosarcina* for which ATP synthesis is dependent on the production and consumption of H_2_ to generate H^+^ gradients^23^. The thermodynamically unfavorable production of H_2_ as an electron transfer agent could impose a barrier to reversing methanogenesis. Metagenomic analyses of the ANME-2a clade, phylogenetically related to *M. acetivorans*, are devoid of hydrogenase genes^21^. Thus, the ANME-2a clade is unlikely to employ H_2_ for transferring electrons to syntrophic partners. It is more likely that electrons are transferred via DIET (direct interspecies electron transfer) as previously hypothesized for uncultured species of the ANME-2 clade^19^. Pathways proposed for ANME include acetate as a product, although disputed as a diffusible electron carrier in syntrophic AOM^12,19,21,32,53^. If not involved in syntrophic AOM, acetate may be an essential carbon source for the syntrophic partner^21^. A portion of acetyl-CoA in the pathway of acetate production (Fig. 5) may also enter biosynthetic pathways essential for AOM by ANME and *M. acetivorans*^54^.

In addition to AOM*, M. acetivorans* is capable of TMO producing CO_2_ or acetate during net methane production when grown with methanogenic substrates in the absence of exogenous electron acceptors^13,14^. Although TMO is likely dependent on reversal of carbon transformation reactions in acetoclastic or CO_2_-reducing methanogenic pathways (Fig. 5), endergonic reactions of TMO are driven by energy conservation dependent on methanogenesis rather than reduction of Fe(III).

## Methods

### Cell growth and materials

*Methanosarcina acetivorans* wild-type strain C2A, isolated from marine sediment, was grown with acetate or methanol as previously described^55,56^. The CO dehydrogenase/acetyl-CoA synthase (CODH/ACS) was purified from acetate-grown cells as previously described^43^. The ferredoxin (Fdx) upregulated in acetate-grown *M. acetivorans* was heterologously produced and purified as previously described^43^. Purification of F_420_ from methanol-grown cells and preparation of F_420_H_2_ was as described elsewhere^57^. AQDS was purchased from ACROS Organics and HSCoB from Tocris Bioscience. CoMS-SCoB heterodisulfide was prepared as described elsewhere^57^. HSCoM, THF and CH_3_-THF were purchased from Sigma Chemical.

Membrane fragments were isolated as described previously^27^. Washed everted membrane vesicles were prepared as described elsewhere except 5 mM ferric citrate or 5 mM AQDS were included in buffers prior to French pressure cell lysis to load vesicles with either or both electron acceptor where indicated^25,57^. The integrity of vesicles was determined by artificial energization as described elsewhere^30^. Maximum quenching of acridine orange was within 5 s followed by 37% dequenching over a period of 4 min.

### Activity assays

All activity assays were performed anaerobically at 21 °C in serum-stoppered glass vials with the indicated gas atmosphere. Pre-reduced Fdx was prepared by incubation with CODH/ACS in 1.0 Atm CO followed by replacement of CO with Ar. The same procedure was used for assays requiring continuous reduction of Fdx except the CO was present throughout the assay. Additions and samplings were performed with gas-tight syringes. Contents of reaction mixtures are included in Figure captions.

The reduced ferredoxin (Fdx_R_):Fe(III) oxidoreductase activity of vesicles was monitored spectrophotometrically following the oxidation of pre-reduced Fdx. Activity was based on the change of absorbance at 410 nm (ε = 30.0 mM^−1^ cm^−1^). Measurement of Na^+^ translocation was as previously described with the following modifications^25^. The reaction mixture was incubated in 1.0 Atm CO for 40 min to ensure Na^+^ equilibrium prior to addition of CODH/ACS to start the reaction. At the time points indicated, the external ^22^Na was removed from samples (100 μl) using a column (0.5 × 3 cm) of DOWEX 50WX8 (Sigma). The vesicles were eluted with 1.0 ml of 0.4 M sucrose followed by addition of 9.0 ml of Ultima Gold LLT scintillation fluid (Perkin Elmer) and measurement of radioactivity with a LSC Beckman Coulter Model LS6500 liquid scintillation counter. The Na^+^ concentration of the reaction mixture was determined with a Ross Sure-Flow (Thermo Fisher Scientific) Na^+^-selective combination electrode.

F_420_H_2_:Fe(III) oxidoreductase activity of vesicles was monitored spectrophotometrically following oxidation of F_420_H_2_. Activity was based on the change of absorbance at 420 nm (ε = 41.4 mM^−1^ cm^−1^). Measurement of H^+^ translocation was as described elsewhere^57^ with the following modifications. An Inlab Micro^TM^ (Mettler-Toledo) pH electrode was connected to a EPU353 pH/ISE isoPOD^TM^ (eDAQ) pH meter interfaced to a computer through Pod-Vu^TM^ software (eDAQ) to monitor the pH change with time. Additions of F_420_H_2_ were made with a gas-tight syringe through the rubber stopper. The pH changes were calibrated with standard solutions of KOH.

The forward and reverse CH_3_-THF:HSCoM methyltransferase activities of vesicles were performed as described elsewhere^29^. Forward activity was monitored by detection of thiol group consumption on transfer of the methyl group from CH_3_-THF to HSCoM. Reverse activity was monitored by detection of HSCoM production on transfer of the methyl group of CH_3_-SCoM to THF.

Reverse heterodisulfide reductase activity (HSCoM+HSCoB→CoMS-SCoB+2H^+^) was assayed as previously described except activity was monitored by consumption of thiol groups^58^.

### Analytical

Production or consumption of HSCoM and HSCoB was determined with Ellman’s reagent^59^. Determination of Fe(II) was by the ferrozine method described elsewhere^60^. Vesicles were collected from the reaction mixture by centrifuging at 135,000 × *g* for 60 min. CHAPS (15 mM) was added to dissolve vesicles and release Fe(II) prior to analysis. Protein was estimated with the Bradford assay kit (Bio-Rad Laboratories) using bovine serum albumin as the protein standard^61^.

### Data availability

All relevant data are available from the authors.

## Electronic supplementary material

Supplementary Information
