# Supplementary material for: A biochemical framework for anaerobic oxidation of methane driven by Fe(III)-dependent respiration
Source: Nat Commun. 2018 Apr 24;9:1642. doi: 10.1038/s41467-018-04097-9 (PMC5915437; doi:10.1038/s41467-018-04097-9)
Supplement: Supplementary file 1 — Supplementary Information [file 41467_2018_4097_MOESM1_ESM.pdf]

Supplementary Information

A biochemical framework for Fe(III)-dependent anaerobic oxidation of methane

Yan *et al.*

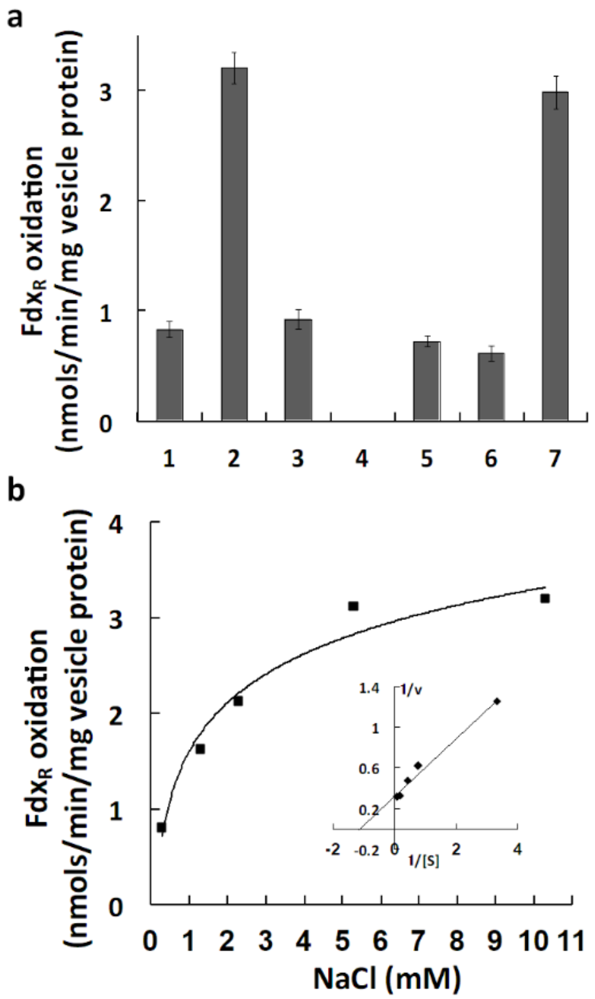

Supplementary Figure 1. Fdx$_R$:Fe(III) oxidoreductase activity of everted membrane vesicles. The standard reaction mixture (0.5 ml) contained 20.5 μM pre-reduced ferredoxin (Fdx$_R$) and 10 mM NaCl in 50 mM Tris (pH 8.0) and 1.0 atmosphere of Ar. Reactions were initiated by addition of Fe(III)-loaded vesicles (0.05 mg protein) prepared from acetate-grown cells. Oxidation of Fdx$_R$ was monitored at 410 nm. **a** Initial velocity of Fdx$_R$ oxidation in standard reaction mixtures modified as indicated for each bar: (1) minus NaCl, (2) standard, no modifications, (3) minus NaCl, plus 10 mM KCl, (4) empty vesicles, (5) Fe(III)-loaded vesicles from methanol-grown cells, (6) 9,10-anthraquinone-2,6-disulfonate (AQDS)-loaded vesicles, (7) AQDS- plus Fe(III)-loaded vesicles. Bars are the mean of three biological replicates for which error bars are the standard deviation. **b** Initial velocity of Fdx$_R$ oxidation in the standard reaction mixture dependent on the concentration of NaCl. Inset, double-reciprocal plot.

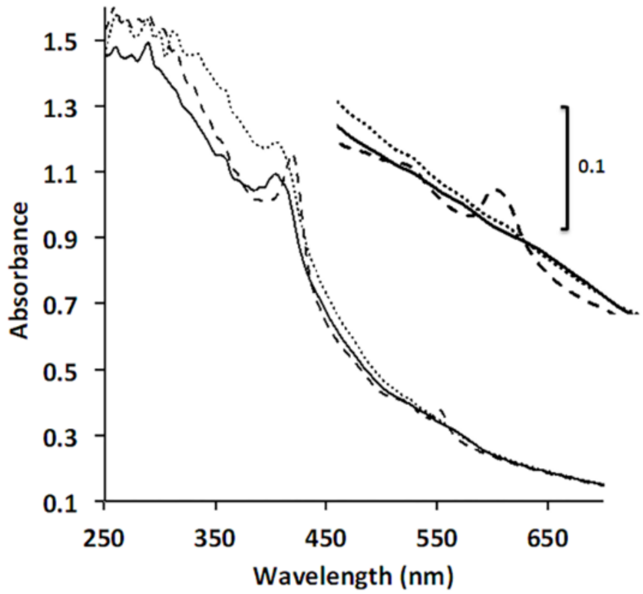

Supplementary Figure 2. UV-Vis spectrum of cytochrome *c* in membranes from acetate-grown *M. acetivorans* reduced with ferredoxin and re-oxidized with Fe(III). Isolated membrane fragments (0.1 mg protein) were contained in 0.5 ml 50 mM Tris (pH 8.0) and 1.0 atmosphere of Ar. As isolated (solid line); after addition of 2.5 μM pre-reduced ferredoxin (Fdx_R) to as-isolated (dash line); after addition of 10 μM ferric citrate to Fdx_R-reduced (dotted line). Inset, spectrum enlarged between 500 and 600 nm. Reduction by Fdx_R is documented by the increased absorbance at 550 nm and shift of the 405 nm peak to 407 nm. Spectra were recorded with a CARY 50 Bio UV-Vis spectrophotometer.

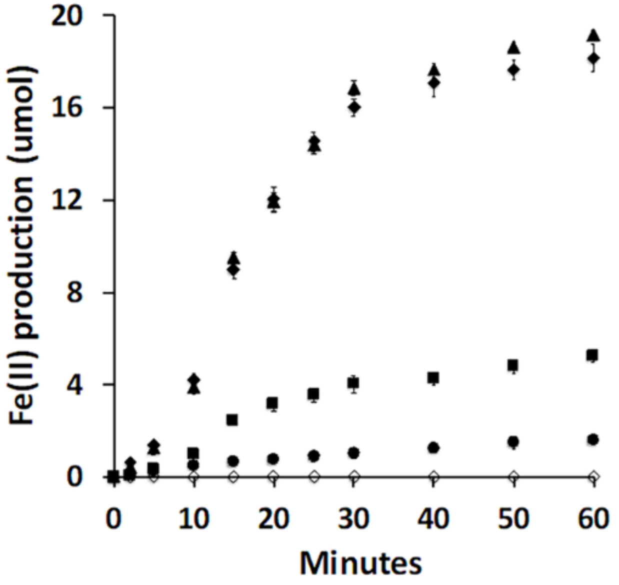

Supplementary Figure 3. Reduction of ferric citrate and ferrihydrite by resting cell suspensions of *M. acetivorans*. The reaction mixture (5 ml) contained 0.25 g (wet weight) acetate-grown cells and 0.5 mM 9,10-anthraquinone-2,6-disulfonate (AQDS) where indicated in 50 mM MOPS-Na buffer (pH 7.0) and 0.1 atmosphere CO. The reaction was started by adding 5 mM ferric citrate or ferrihydrite. Ferrihydrite was prepared as described elsewhere[1]. Symbols: (▲) ferric citrate plus AQDS, (◆) ferric citrate, (■) ferrihydrite plus AQDS, (●) ferrihydrite, (◇) minus electron acceptor or 1.0 atmosphere N_2. Data points are the mean of three biological replicates for which the standard deviation is shown by error bars.

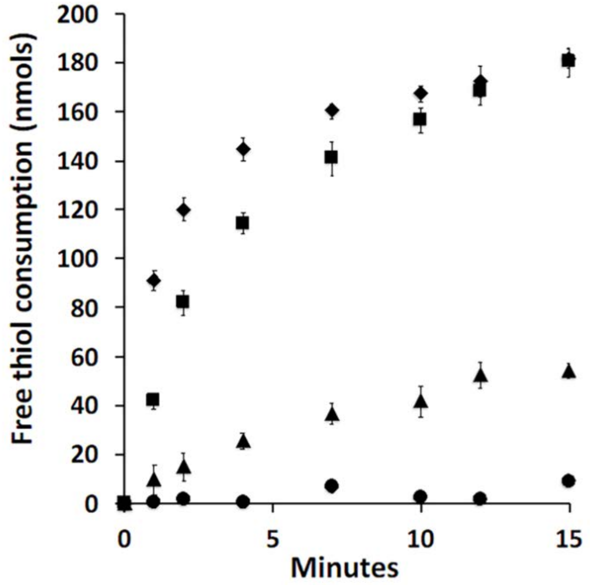

Supplementary Figure 4. Methyltransferase activity in the direction of CH_3-SCoM production. The standard reaction mixture (1.0 ml) contained 3.0 mM coenzyme M (HSCoM), 1.5 mM methyl-tetrahydrofolate (CH_3-THF), 2 mM ATP, 0.4 M sucrose, 20 mM $MgSO_4$, 1.0 mM dithiothreitol and empty vesicles (0.5 mg protein) from acetate-grown cells in 50 mM MOPS buffer (pH 7.0) in 1.0 atmosphere of CO. Symbols: (◆) standard reaction mixture, (■) minus CO, (▲) minus ATP, (●) minus HSCoM or CH_3-THF. Data points are the mean of three biological replicates for which the standard deviation is shown by error bars.

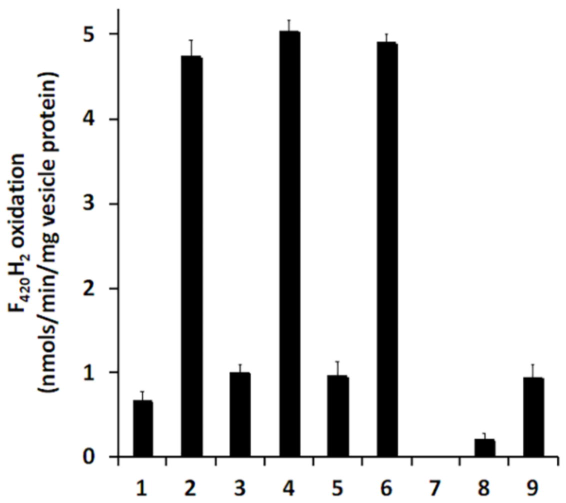

Supplementary Figure 5. $F_{420}H_2$:Fe(III) oxidoreductase activity of everted membrane vesicles. The standard reaction mixture (0.5 ml) contained 12.5 μM reduced coenzyme F_{420} ($F_{420}H_2$) in 50 mM Tris pH 8.0 and 1.0 atmosphere Ar. Reactions were initiated by addition of Fe(III)-loaded everted vesicles (50 μg protein) from methanol-grown cells. Values shown are initial rates of $F_{420}H_2$ oxidation monitored at 420 nm with a CARY 50 Bio-UV-Vis spectrophotometer. Bars are the mean of three biological replicates for which the standard deviation is shown by error bars. Standard reaction mixtures were modified as indicated: (1) no modification, (2) plus 2.0 μM carbonyl cyanide m-chlorophenyl hydrazine (CCCP), (3) 9,10-anthraquinone-2,6-disulfonate (AQDS)-loaded vesicles, (4) AQDS-loaded vesicles plus 2.0 μM CCCP, (5) AQDS- and Fe(III)-loaded vesicles, (6) AQDS- and Fe(III)-loaded vesicles plus 2.0 μM CCCP, (7) empty vesicles, (8) Fe(III)-loaded vesicles from acetate-grown cells, (9) Fe(III)-loaded vesicles from acetate-grown cells plus 2.0 μM CCCP.

Enzyme (reaction Number)*	Reaction	$\Delta G^{\circ\prime}$ (kJ)
Mcr (1)	5 x (CH_4 + CoMS-SCoB → CH_3-SCoM + HSCoB)	5 x (+45.0)[a]
Mtr (2)	5 x (CH_3-SCoM + H_4SPT → CH_3-H_4SPT + HS-CoM)	5 x (+30.0)[a]
Rnf (3)	5 x (Fdx_R+ $2Fe^{3+}$ → $2Fe^{2+}$ + Fdx_O)	5 x (-249.3)[b]
HdrDE (4)	5 x (HS-CoM + HS-CoB + $2Fe^{3+}$ → CoMS-SCoB + $2Fe^{2+}$+ $2H^+$)	5 x (-175.8)[c]
CODH/ACS (5)	CH_3-H_4SPT + CO_2 + HS-CoA + Fdx_R + $2H^+$ → CH_3-SCoA + H_4SPT + H_2O + Fdx_O	-40.5[d]
Pta, Ack (6)	CH_3-CO-SCoA + H_2O → HS-CoA + CH_3COOH	-35.7[a]
Mer, Mtd, Mch (7)	4 x (CH_3-H_4HPT + $2F_{420}$ → CHO-H_4HPT + $2F_{420}H_2$)	4 x (-5.3)[a]
Ftr, Fmd (8)	4 x (CHO-H_4HPT + Fdx_O + H_2O → Fdx_R + H_4HPT + CO_2 + H^+)	4 x (-32.0)[d]
Fpo (9)	4 x ($F_{420}H_2$ + $2Fe^{3+}$ → $F_{420}H_2$ + $2Fe^{3+}$ + $2H^+$)	4 x (-222.3)[e]
HdrA2B2C2 (10-12)	2 x ($2F_{420}H_2$ + $2Fdx_O$ + CoMS-SCoB → $2F_{420}$ + $2Fdx_R$ + HSCoM + HSCoB)	2 x (-38.5)[f]
HdrDE (13)	2 x (HS-CoM + HS-CoB + $2Fe^{3+}$ → CoMS-SCoB + $2Fe^{2+}$+ $2H^+$)	2 x (-175.8)[c]
Atp (14)	13 x (ADP + HPO_4^{-}→ATP + H_2O)	13 x (+31.8)[a]
Overall	5 CH_4 + $32Fe^{3+}$ + 13ADP + $13HPO_4^{-}$ → CH_3COOH + $32Fe^{3+}$ + $19H^+$ + 13ATP + $3CO_2$ + $5H_2O$	-2,880.3 kJ

*See Figure 5 for corresponding reaction numbers. Mcr, methyl-coenzyme M reductase; Mtr, methyltransferase; HdrDE, membrane-bound heterodisulfide reductase; Cyt c, multi-heme cytochrome c; CODH/ACS, CO dehydrogenase/acetyl-CoA synthase; Pta, phosphotransacetylase; Ack, acetate kinase; Mer, F_{420}-dependent methylene-H_4MPT reductase; Mtd, F_{420}-dependent methylene-H_4MPT dehydrogenase; Mch, methenyl-H_4MPT cyclohydrolase; Ftr, formylmethanofuran:H_4MPT formyltransferase; Fmd, formylmethanofuran dehydrogenase; HdrA2B2C2, cytoplasmic heterodisulfide reductase; Rnf, Rnf complex.
[a]Published values[2,3].
[b]Calculated with published standard midpoint potentials (+772 mV for Fe^{3+}/Fe^{2+} and -520 mV for Fdx_O/Fdx_R^{2-}) using $\Delta G = -nF\Delta E$ and the Faraday constant of 0.09648 kJ/eV[3].
[c]Calculated with published standard midpoint potentials (+772 mV for Fe^{3+}/Fe^{2+} and -140 mV for CoMS-SCoB/HSCoM + HSCoB) using $\Delta G = -nF\Delta E$ and the Faraday constant of 0.09648 kJ/eV[3,4].
[d]Published value[2].
[e]Calculated with standard midpoint potentials (-520 mV for Fdx_O/Fdx_R^{2-} and -380 mV for $F_{420}/F_{420}H_2$) using $\Delta G = -nF\Delta E$ and the Faraday constant of 0.09648 kJ/eV.
[f]Calculated with published standard midpoint potentials (-520 mV for Fdx_O/Fdx_R^{1-}, -140 mV for CoMS-SCoB/HSCoM + HSCoB and -380 mV for $F_{420}/F_{420}H_2$) using $\Delta G = -nF\Delta E$ and the Faraday constant of 0.09648 kJ/eV.

Supplementary Table 1. Standard Gibbs free energy values for reactions in the Fe(III)-dependent ANME pathway proposed for *Methanosarcina acetivorans*.

ANME-2a		Query		Subject			Identities	*M. acetivorans*	
Gene Id	Gene Name	Start Coordinate	End Coordinate	Start Coordinate	End Coordinate	Length		loci	length
2566124572	electron transport complex protein RnfB	3	263	5	264	274	61	RnfB MA0663	264
2566124573	electron transport complex protein RnfA	4	197	1	193	194	61	RnfA MA0663	199
2566124577	electron transport complex protein RnfC	17	432	9	403	441	39	RnfC MA0659	447
2566124576	electron transport complex protein RnfD	3	285	2	291	293	46	RnfD MA0660	288
2566124574	electron transport complex protein RnfE	7	213	2	206	207	68	RnfE MA0662	213
2566124575	electron transport complex protein RnfG	4	186	2	176	176	44	RnfG MA0661	188
2566124578	multiheme c-type cytochrome	24	494	20	475	477	36	CytC MA0658	500
2566123507	F420H2 dehydrogenase subunit A	1	124	1	132	132	54	FpoA MA1495	124
2566123508	F420H2 dehydrogenase subunit B	21	180	17	176	187	69	FpoB MA1496	184
2566123510	F420H2 dehydrogenase subunit D	6	374	13	376	376	58	FpoD MA1498	374
2566123511	F420H2 dehydrogenase subunit H	13	345	19	348	350	57	FpoH MA1499	348
2566123512	F420H2 dehydrogenase subunit I	1	125	1	115	131	47	FpoI MA1500	136
2566123513	F420H2 dehydrogenase subunit J	30	88	23	81	85	54	FpoJ MA1501	96
2566123515	F420H2 dehydrogenase subunit K	4	102	2	100	100	60	FpoK MA1503	102
2566123516	F420H2 dehydrogenase subunit L	5	666	2	676	681	59	FpoL MA1504	672
2566123518	F420H2 dehydrogenase subunit N	8	488	11	490	492	56	FpoN MA1506	489
2566123509	F420H2 dehydrogenase subunit C	1	153	1	150	150	55	FpoC MA1507	158
2566123517	F420H2 dehydrogenase subunit M	4	486	5	488	493	55	FpoM MA1505	495
2566126466	heterodisulfide reductase, subunit D	1	409	11	435	459	55	HdrD MA0688	409
2566126467	heterodisulfide reductase, subunit E	1	264	1	248	248	43	HdrE MA	264
2566125857	methyltransferase, subunit A	1	224	1	224	242	63	MtrA MA0272	240
2566125858	methyltransferase, subunit B	1	108	1	104	104	43	MtrB MA0273	108
2566125859	methyltransferase, subunit C	7	267	6	284	284	54	MtrC MA0274	267
2566125860	methyltransferase, subunit D	10	240	11	245	252	61	MtrD MA0275	249
2566125861	methyltransferase, subunit E	4	304	6	301	301	61	MtrE MA0276	304
2566125856	methyltransferase, subunit F	7	73	46	113	115	50	MtrF MA0271	74
2566125855	methyltransferase, subunit G	1	65	1	65	86	51	MtrG MA0270	73
2566125854	methyltransferase, subunit H	1	315	1	311	312	73	MtrH MA0269	316
2566123466	acetyl-CoA decarbonylase/synthase alpha subunit	4	805	3	791	793	59	CdhA MA3860	805
2566123467	acetyl-CoA decarbonylase/synthase epsilon subunit	2	170	6	176	176	46	CdhB MA3861	170
2566123468	acetyl-CoA decarbonylase/synthase beta subunit	3	467	4	466	466	66	CdhC MA3862	470
2566123469	CO dehydrogenase maturation factor	3	252	2	248	249	57	CdhD MA3863	253
2566123471	acetyl-CoA decarbonylase/synthase delta subunit	1	436	1	434	434	69	CdhE MA3864	436

2566125579	methyl-coenzyme M reductase, alpha subunit	9	570	16	575	575	69	McrA 4546	570
2566125583	methyl-coenzyme M reductase, beta subunit	1	434	1	434	434	66	McrB 4550	434
2566125580	methyl-coenzyme M reductase, gamma subunit	1	248	1	248	248	77	McrG 4547	248

SupplementaryTable 2. Sequence identities for proteins in pathways proposed for *Methanosarcina acetivorans* and uncultured ANME-2a. The *Methanosarcinales* sp. ANME-2a genome (ID 2565956544) (https://img.jgi.doe.gov/) was queried with protein sequences from *Methanosarcina acetivorans* (https://www-ncbi-nlm-nih-gov).

Supplementary References

1 Yamada, C., Kato, S., Kimura, S., Ishii, M. & Igarashi, Y. Reduction of Fe(III) oxides by phylogenetically and physiologically diverse thermophilic methanogens. *FEMS Microbiol. Ecol.* **89**, 637-645 (2014).

2 Thauer, R. K. Biochemistry of methanogenesis: a tribute to Marjory Stephenson. *Microbiol.* **144**, 2377-2406 (1998).

3 Thauer, R. K., Jungermann, K. & Decker, K. Energy conservation in chemotrophic anaerobic bacteria. *Bacteriol. Rev.* **41**, 100-180 (1977).

4 Thauer, R. K., Kaster, A. K., Seedorf, H., Buckel, W. & Hedderich, R. Methanogenic archaea: ecologically relevant differences in energy conservation. *Nat. Rev. Microbiol.* **6**, 579-591 (2008).
